# Classification of social behavioral responses in stress and non-stress adult male mice with high precision

**DOI:** 10.1038/s44277-025-00047-8

**Published:** 2025-12-03

**Authors:** Jose M. Restrepo-Lozano, Maxime Teixeira, Giovanni Hernandez, Lucas Miranda, Ashraf Mahmud, Mathias V. Schmidt, Cecilia Flores

**Affiliations:** 1 Integrated Program in Neuroscience, McGill University, Montreal, Quebec Canada; 2 Douglas Mental Health University Institute, Montreal, Quebec Canada; 3 International Max Planck Research, School for Translational Psychiatry, 80804 Munich, Germany; 4 Department of Psychiatry, Faculty of Medicine, McGill University, Montreal, Quebec Canada; 5 Research Group Neurobiology of Stress Resilience, Max Planck Institute of Psychiatry, 80804 Munich, Germany; 6 Department of Neurology and Neurosurgery, Faculty of Medicine, McGill University, Montreal, Quebec Canada

**Keywords:** Social behaviour, Stress and resilience

## Abstract

To better characterize social behavior following stress exposure in mice, this study introduces a bidimensional analytical framework that extends beyond the limits of conventional analysis. By integrating a time-based social interaction ratio with the average distance to the CD1 aggressor, we propose a composite index that offers a more comprehensive assessment of social engagement during the social interaction test. This metric distinguishes between socially hesitant male and female mice— those entering the interaction zone while maintaining a relative distance from the CD1 aggressor—and mice that display robust sociability by both entering the zone and closely approaching the aggressor. By treating distance as a continuous variable, this approach moves beyond binary zone-based measures and enables a more refined phenotyping of individual differences along the resilience–susceptibility spectrum. This advancement is made possible using open-source, multipose-estimation tools such as DeepLabCut and DeepOF, which allow for high-resolution tracking and behavioral quantification. Our framework refines current preclinical models by capturing subtle behavioral adaptations to social stress. This ultimately improves their translational value for studying the neural and behavioral correlates of stress-related psychiatric disorders.

## Lay summary

This study developed a new way to analyze how mice behave socially after stress. Instead of just looking at how much time they spend near another mouse, we also measured their distance from that mouse. This helps us identify mice that are hesitant but still engage, versus those that are truly social. Using advanced tracking software, our method provides a more detailed picture of social responses, improving how we study stress-related conditions and their impact on behavior.

## Introduction

Animal models have proven invaluable for probing the complex relationship between stress and psychiatric disorders in both fundamental and translational research [1]. Among these, the Chronic Social Defeat Stress (CSDS) paradigm is particularly notable for its robustness and face validity, effectively capturing behavioral endophenotypes associated with depression and anxiety [2, 3]. The CSDS protocol involves subjecting an experimental mouse to brief, daily episodes of social subjugation by a larger, aggressive conspecific. Following each encounter, the mouse is maintained in protected sensory contact with the aggressor for 24 h, with this cycle typically repeated for ten days. Subsequent assessment with a two-phase social interaction test—where time spent near an enclosure, first empty and then containing a novel aggressor, is measured—allows for the classification of animals into “susceptible” (exhibiting social avoidance) or “resilient” (exhibiting pro-social behavior) [2, 3]. While the CSDS model has significantly advanced our understanding of the neurobiology of stress, the traditional analytical methods have limitations. Specifically, the binary classification of animals as either susceptible or resilient is now recognized as a simplification of the continuum of stress vulnerability and social behavior (for a review, see [4–6]). Relying solely on time spent in a pre-defined “social interaction zone” is an overly reductionist readout that fails to capture the nuanced dynamics of social investigation and avoidance, potentially obscuring meaningful individual differences in stress responsiveness [7]. To overcome these limitations, recent advances in computational tools offer promising alternatives. A key development in behavioral phenotyping is the creation of open-source, machine learning-based tools for markerless body-pose estimation, which have vastly improved the speed and accuracy of data acquisition [8, 9]. Tools such as DeepLabCut [8] —the most popular of its kind— SLEAP [10], DeepPoseKit [11], among many others [12] enable the tracking of multiple user-defined body parts from video recordings. By generating precise, time-resolved data, these deep learning approaches overcome the key constraints of traditional scoring methods and have notably improved the interpretation of rodent behavior in tasks such as social avoidance [13, 14].

Building upon this technological foundation, the Python-based DeepOF framework introduces a novel paradigm for analyzing social behavior [15]. DeepOF enhances the ethological validity of social phenotyping by moving beyond the classic reliance on social interaction time to offer a multi-dimensional analysis of spatiotemporal behavioral patterns. By enabling a detailed classification of individual behavioral profiles, this software expands the analytical power of classic social interaction tasks. As an open-source tool, DeepOF also promotes accessibility, reproducibility, and collaboration within the research community [16, 17].

This approach leverages open-source tools to introduce a bi-dimensional analytical framework that enhances the standard evaluation of the social interaction task. We present a more comprehensive and unbiased view of how stress exposure shapes social behavior in adult male and female mice, enhancing the detection of individual differences and supporting better extrapolations to behavioral traits relevant to stress-related psychiatric disorders.

## Materials and methods

Experimental procedures were performed in accordance with the guidelines of the Canadian Council of Animal Care and were approved by the McGill University and Douglas Hospital Animal Care Committee. All CD-1 mice used in these studies were obtained from Charles River Canada and all C57BL/6 J were obtained from Jackson Laboratories  and maintained on a 12 h light-dark cycle (light on at 8:00 h) with *ad libitum* access to food and water throughout the experiments. All experiments were conducted during the light part of the cycle.

### Animals

**Fig. 1:**
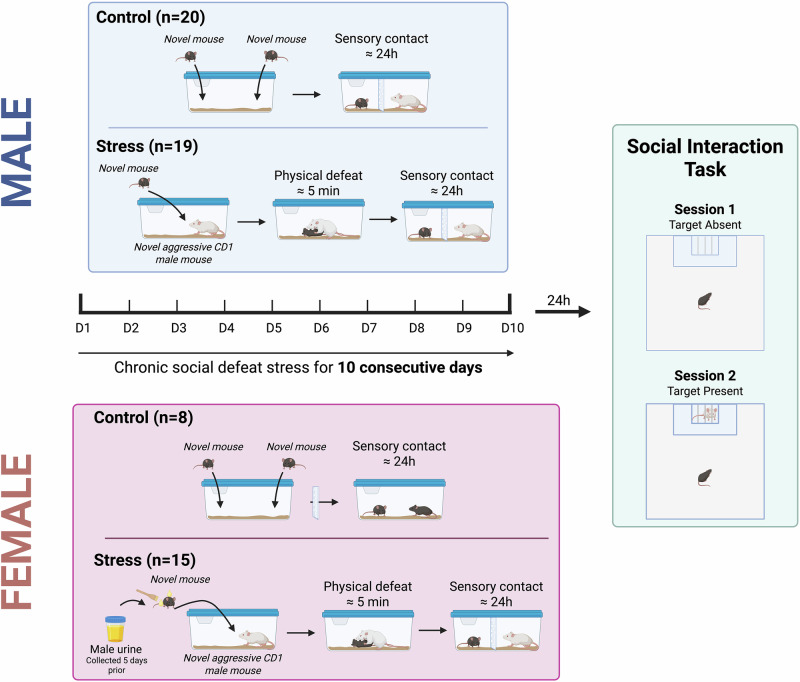
Experimental design for male and female mice and timeline. The timeline illustrates the procedure for inducing CSDS in experimental mice over 10 consecutive days (D1-D10). *Male:* For the control group (n = 20), each mouse was introduced to a novel conspecific in a two-compartment cage with sensory but not physical contact for approximately 24 h. The stress group (n = 19) underwent a CSDS regimen, where each mouse was subjected to physical aggression from a novel CD-1 aggressor mouse. Post-aggression, mice were housed in two-compartment cages providing sensory contact with the aggressor for 24 h. Each day featured a new CD-1 aggressor for the stressed mice. *Female*: In adult female mice, CSDS was conducted similarly to the male protocol for control mice (n = 8), with a few modifications for the stress group (n = 15). Specifically, five days prior to the start of CSDS, urine was collected from adult C57BL/6 J male mice. During each defeat session, urine was applied to the base of the tail and vaginal area using a paintbrush, just before placing the female into the home cage of an aggressive CD-1 mouse. The two groups were subsequently assessed for social interaction in a two-session task, with the second session introducing a “social” target.

C57BL/6 J wild-type male and female (PD 90 ± 15) mice served as experimental subjects in the CSDS paradigm. Mice were randomly assigned to stress (n = 19 males, 15 females) or non-stress (n = 20 males, 8 females) conditions (Fig. 1). At the completion of the last defeat session, mice were housed individually prior to conducting the social interaction task to measure social avoidance. Male CD-1 retired breeder mice (less than 3 months old and previously screened for aggressive behavior) were used as social aggressors. CD-1 mice were single-housed throughout the study. All behavioral experiment were run in the morning, typically between 10:00 to 12:00

#### Chronic social defeat stress

CSDS in adult male mice was performed as described in [2]. Briefly, experimental adult male mice were exposed to 5 min of physical aggression (or to a total of 10 attacks) as per the guidelines of McGill standard operating procedure 416 (SOP416) by a novel CD-1 mouse, previously screened for aggressive behavior. Upon session completion, both experimental mouse and CD1 aggressor were housed overnight, separated by a transparent divider providing sensory but not physical contact. The procedure was repeated for a total of 10 consecutive days (as per SOP416), in which all experimental mice faced a new aggressor every day. Control mice were housed in pairs in the defeat boxes in a different room. Each control mouse was placed on one side of the perforated divider. Like the mice undergoing defeat, all control mice were rotated daily, however they never had physical contact with their cage mate [2]. Twenty-four hours after the last CSDS session, mice were evaluated in the social interaction task.

In adult female mice, CSDS was performed in a similar fashion as in males but with a few modifications, according to previous studies [18, 19]: five days prior to CSDS, urine from adult C57BL/6 J male mice was collected and stored at 4 °C. Throughout the defeat sessions, each female mouse was applied urine from an adult C57BL/6 J male mice. The urine was placed with a paintbrush to the base of the tail and in vaginal area of the female mice before they were immediately placed in an aggressor CD-1 mouse’s home cage for a 5-minute (or up to 15 attacks, whichever comes first). Other aspects of the defeat process for females were identical to the male CSDS.

#### Social interaction task

**Fig. 2:**
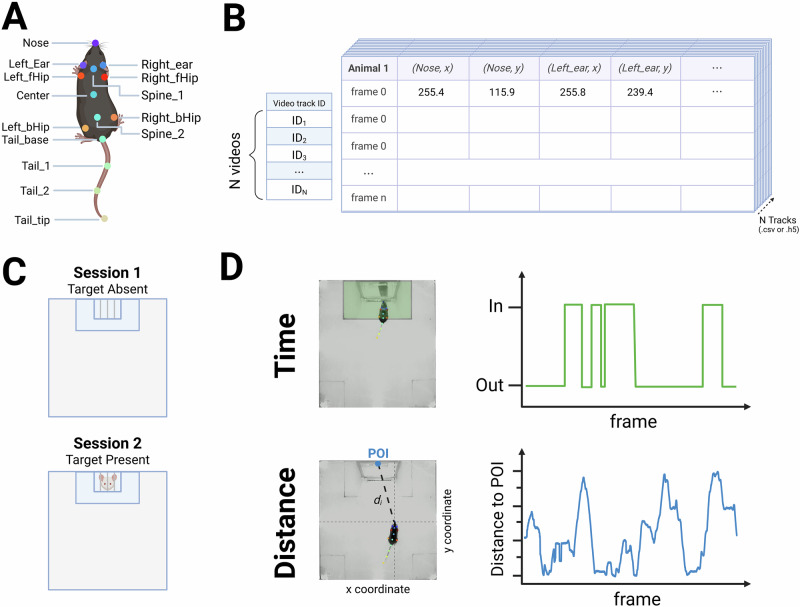
Overview of behavioral tracking and quantification methods. **A** Example of DeepLabCut (DLC) tracking labels applied to individual mice for time-series motion capture. **B** Representative output of DLC tracking, showing track IDs and associated body part coordinates. **C** Schematic of the classic Social Interaction Task, which includes two sessions: Session 1 (social target absent) and Session 2 (social target present). The diagram illustrates the social interaction zone (SIZ, light blue). **D** Overview of the two behavioral quantification methods used in the chronic social defeat paradigm. *Top*: a binary measure based on time spent within the SIZ. *Bottom*: a continuous measure based on the distance between a defined point of interest (POI; defined as the midpoint between the left and right top corners of the cage) and the experimental mouse’s nose.

As described previously [2], the task consisted of 2 sessions in which experimental and control mice were allowed to explore a square arena (42 × 42 cm) in the absence (Session 1) or presence (Session 2) of a novel CD-1 mouse contained in a cage, for a period of 2.5 min per session. Measurements of the total time spent by the mouse within the boundaries of the social interaction zone (SIZ) with and without a novel CD-1 adult male mouse, were recorded. To enrich our behavioral analysis, we also generated a novel metric assessing the distance between a point of interest and the mouse’s nose (Fig. 2).

#### Nest building test

For female mice, 3 h after the last CSDS session, all mice were evaluated in the nest building test – a measure of self-care-like behaviors [20, 21]. Specifically, a single nestlet was placed into the home cage of each mouse and 3 h later, mice were scored for nest building using a 5-point nest rating scale, as before (1 = nestlet not noticeably touched; 2 = nestlet partially torn; 3 = nestlet mostly shredded but no clear nest site; 4 = nest with identifiable structure but flat; 5 = A well-structured, dome-shaped nest covering the mouse) [20, 21].

### Animal tracking and time series motion tracks

All videos were analyzed with DeepLabCut version 2.2.2 (*see* Supplementary Fig. 2), tailored for DeepOF bodypart_graph identification (deepof_14), to generate the time-series motion tracks from each social interaction task video – recorded at 30 frames per second – which were further processed using DeepOF v0.6. Custom Python scripts were used to compute distances to a point of interest, total distance traveled, and total time within the social interaction zone. These scripts are publicly accessible for replication and further analysis (github.com/madmaxpython/DeepOF_SIT).

### Calculating social interaction ratios

#### Time-based social interaction ratios

For each individual mouse, we determined whether the center of its body was located within the social interaction zone during Session 1 (CD-1 absent) and Session 2 (CD-1 present), as shown in Fig. 2C.

Time-based social interaction ratios were calculated using formulas (1) and (2):

1 $${{SIR}}_{{typeA}}=\frac{\frac{1}{n}\,\mathop{\sum }_{i}^{n}{Time\; in\; SIZ}({session} \, 2)\,}{\frac{1}{n}\,\mathop{\sum }_{i=0}^{n}{Time\; in\; SIZ}({session} \, 1)}$$

2 $${{SIR}}_{{typeB}}=\frac{\frac{1}{n}\,\mathop{\sum }_{i=0}^{n}{Time} \; {in} \; {SIZ}({session} \, 2)\,}{\frac{1}{n}\,\mathop{\sum }_{i=0}^{n}{Time} \; {in} \; {SIZ}\left({session} \, 2\right)+\,\frac{1}{n}\mathop{\sum }_{i=0}^{n}{Time} \; {in} \; {SIZ}({session} \, 1)}$$

Defeated mice were classified as susceptible or resilient based on these ratios, with susceptibility defined by a SIR_typeA_ $<$ 1 or SIR_typeB_ $<$ 0.5, and resilience by SIR_typeA_ $\ge \,$1 or SIR_typeB_ $\ge \,$0.5.

#### Distance-based Social Interaction Ratios

As shown in Fig. 2, we measured the distance of mice to a point of interest (POI) corresponding to the cage in the social interaction zone, empty in Session 1 and containing a CD1 aggressor mouse in Session 2. This point was defined as the midpoint between the left and right top corners of the cage. Distances were converted from pixels to meters for consistency. Distance-based social interaction ratios were calculated using formulas (3) and (4) focusing on the TypeB formulas for classifying mice in this study:

3 $${{SIR}}_{{typeA}}=\frac{\frac{1}{n}\,\mathop{\sum }_{i}^{n}{Distance}{\_}{to}{\_}{POI}({session} \, 2)\,}{\frac{1}{n}\,\mathop{\sum }_{i=0}^{n}{Distance}{\_}{to}{\_}{POI}({session} \, 1)}$$

4 $${{SIR}}_{{typeB}}=\frac{\frac{1}{n}\,\mathop{\sum }_{i=0}^{n}{{Distance}{\_}{to}{\_}{POI}}_{i}\,({session} \, 2)\,}{\frac{1}{n}\,\mathop{\sum }_{i=0}^{n}{Distance}{\_}{to}{\_}{POI}\left({session} \, 2\right)+\,\frac{1}{n}\mathop{\sum }_{i=0}^{n}{Distance}{\_}{to}{\_}{POI}({session} \, 1)}$$

#### Social Engagement Index

We proposed a new index combining time- and distance-based measures of interaction to better capture social engagement in mice. Calculated using formula (5), this metric is more sensitive to subtle differences in social behavior, distinguishing a “hesitantly social” mouse (one that enters the zone but keeps its distance) from a “robustly social” one (that both enters the zone and approaches closely).

5 $${Inde}{x}_{{Social}{\_}{engagement}}=\frac{{Time}{\_}{SI}{R}_{{TypeB}}\,}{{Distance}{\_}{SI}{R}_{{TypeB}}}\,$$

A high index value indicates an increased time in the interaction zone (large numerator) and a low distance from the point of interest (small denominator). This high index value can be interpreted as a strong social approach, probably reflecting low social avoidance. On the other hand, a low index value indicates a decreased time in the interaction zone (small numerator) and high distance from the point of interest (large denominator). This low index value can be interpreted as a weak social approach, reflecting high social avoidance.

### Statistical analysis

Statistical analyses and graphs were generated in GraphPad Prism and Python (v 3.10). All outcome variables were tested for normality using the Shapiro-Wilk test and homogeneity of variance using Levene’s test. Two-way repeated measures ANOVA was used to assess the effect of session (within-subject factor) across conditions. One-way ANOVA was applied for three-group comparisons. When appropriate, post-hoc analyses were performed using the Tukey’s HSD or Sidak’s test to identify pairwise differences. To assess if the social behavioral phenotypes could be distinguished based on two continuous parameters (i.e. distance and time-based metrics), we conducted a multivariate analysis of variance (MANOVA). We performed a Wilks’ Lambda test to evaluate overall multivariate group differences. Spearman’s rank correlation was used to analyze the association between the Social Engagement Index and the nest building test score (treated as a categorical variable). Statistical significance was defined as *p* < 0.05 (*), with *p* < 0.01 (**), *p* < 0.001 (***), *p* < 0.0001 (****). Detailed information about all statistical tests is presented in Supplementary Tables 1–3.

## Results

### Spatiotemporal social interaction ratios are clear metrics of social impairment in male and female mice

#### Social phenotype description

To assess social interaction phenotypes in mice, we employed two complementary metrics: the total time spent in the social interaction zone (SIZ), and the average distance from a defined point of interest (POI). While time in the SIZ is a widely established measure of social approach, we introduced distance to the POI as a novel, continuous metric that reduces reliance on subjectively defined spatial boundaries and captures behavioral nuances potentially overlooked by time-based measures alone.

#### Social interaction behavior in male mice

**Fig. 3:**
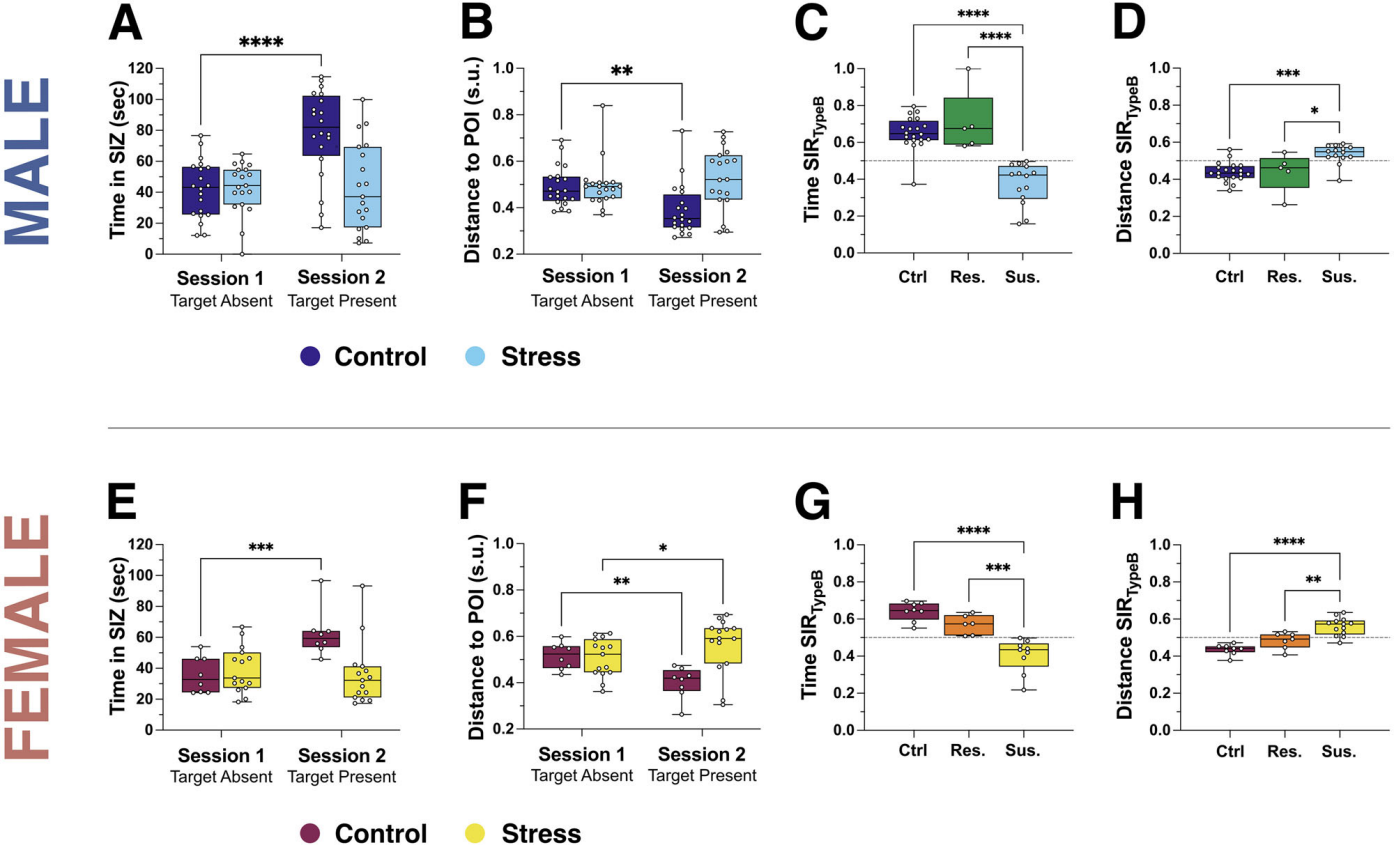
A spatiotemporal social interaction ratio quantifies social impairment in male and female mice. **A**, **B** In male mice, control animals spent more time in the Social Interaction Zone (SIZ) during Session 2 – social target present – compared to Session 1 – target absent – (**A**) and showed reduced distance to the Point of Interest (POI)(**B**) (Supplementary Table 1A, B). In contrast, CSDS-exposed males showed no significant change across Sessions for either metric, suggesting a stress-induced disruption of social approach behavior. **C**, **D** Social Interaction Ratios (SIRTypeB) in males, based on time (**C**) and distance (**D**), revealed that susceptible animals displayed lower time-based SIRs and higher distance-based SIRs compared to both control and resilient mice (Supplementary Table 1C, D), consistent with a socially avoidant phenotype. **E**, **F** In female mice, control animals similarly increased time in the SIZ and decreased distance to the POI during Session 2, relative to Session 1 (Supplementary Table 1E, F). In contrast, CSDS-exposed females showed no change in SIZ time and a modest increase in distance to the POI, consistent with social withdrawal. **G**, **H** Time-based (**G**) and distance-based (**H**) SIRTypeB measures in females showed reduced social interaction in susceptible animals compared to control and resilient groups (Supplementary Table 1G, H), mirroring findings in males and supporting the validity of both metrics in capturing stress-induced social avoidance. Box plots represent the median (line), first and third quartiles (box), and minimum/maximum values (whiskers). *p < 0.05, *p < 0.01, **p < 0.001, ***p < 0.0001.

We first examined behavior in male mice following CSDS or control conditions. Control male mice showed a significant increase in time spent in the SIZ during Session 2 – when the CD-1 mouse was present – compared to Session 1 – when it was absent – reflecting normal social approach (Fig. 3A). In contrast, CSDS-exposed males failed to increase their time in the SIZ across sessions, indicating a disruption in social motivation consistent with a social avoidance phenotype.

We next assessed proximity to the POI in male mice. Control mice showed a significant reduction in distance to the POI in Session 2 (Fig. 3B), indicating approach behavior toward the social target). However, CSDS-exposed males did not show any change in proximity between Sessions, further supporting the presence of social avoidance in this group.

To quantify individual variability in stress susceptibility, we applied the conventional time-based social interaction ratio (SIRTypeB, see Eq. 2 in Method section) and stratified CSDS-exposed males into susceptible (SIR < 0.5) and resilient (SIR $\ge$ 0.5) phenotypes. A significant main effect of the phenotype was observed, with susceptible males showing significantly lower time-based SIR than both control and resilient mice (Fig. 3C).

Using the same classification, we assessed the distance-based SIRTypeB (see Eq. 4 in Method section). Susceptible males showed a significantly higher distance-based SIR than both control and resilient animals (Fig. 3D), validating this novel metric as a reliable proxy for social avoidance in male mice.

#### Social interaction behavior in female mice

We then applied the same analyses to female mice. Control females spent significantly more time in the SIZ during Session 2 compared to Session 1 (Fig. 3E), consistent with preserved social approach. However, CSDS-exposed females did not show this increase in SIZ time, indicating impaired social engagement following stress exposure, as observed in males.

Analysis of POI proximity in female mice revealed a significant decrease in distance between Sessions in the control group (Fig. 3F), consistent with approach toward the social target. In contrast, CSDS-exposed females displayed a significant increase in distance to the POI in Session 2 relative to Session 1, highlighting a pronounced social avoidance phenotype in stressed females.

Time-based SIR values were then used to classify CSDS-exposed females as susceptible or resilient. As in males, a significant the phenotype effect was observed, with susceptible females displaying significantly lower time-based SIR than both controls and resilient animals (Fig. 3G).

When we assessed the distance-based SIR using the same classification, susceptible females again showed significantly higher values than both control and resilient groups (Fig. 3H). This further confirms that the distance-based SIR reliably tracks social avoidance in both sexes and parallels findings from the traditional time-based metric.

### Combining time and distance metrics unmasks individual differences in social interaction

**Fig. 4:**
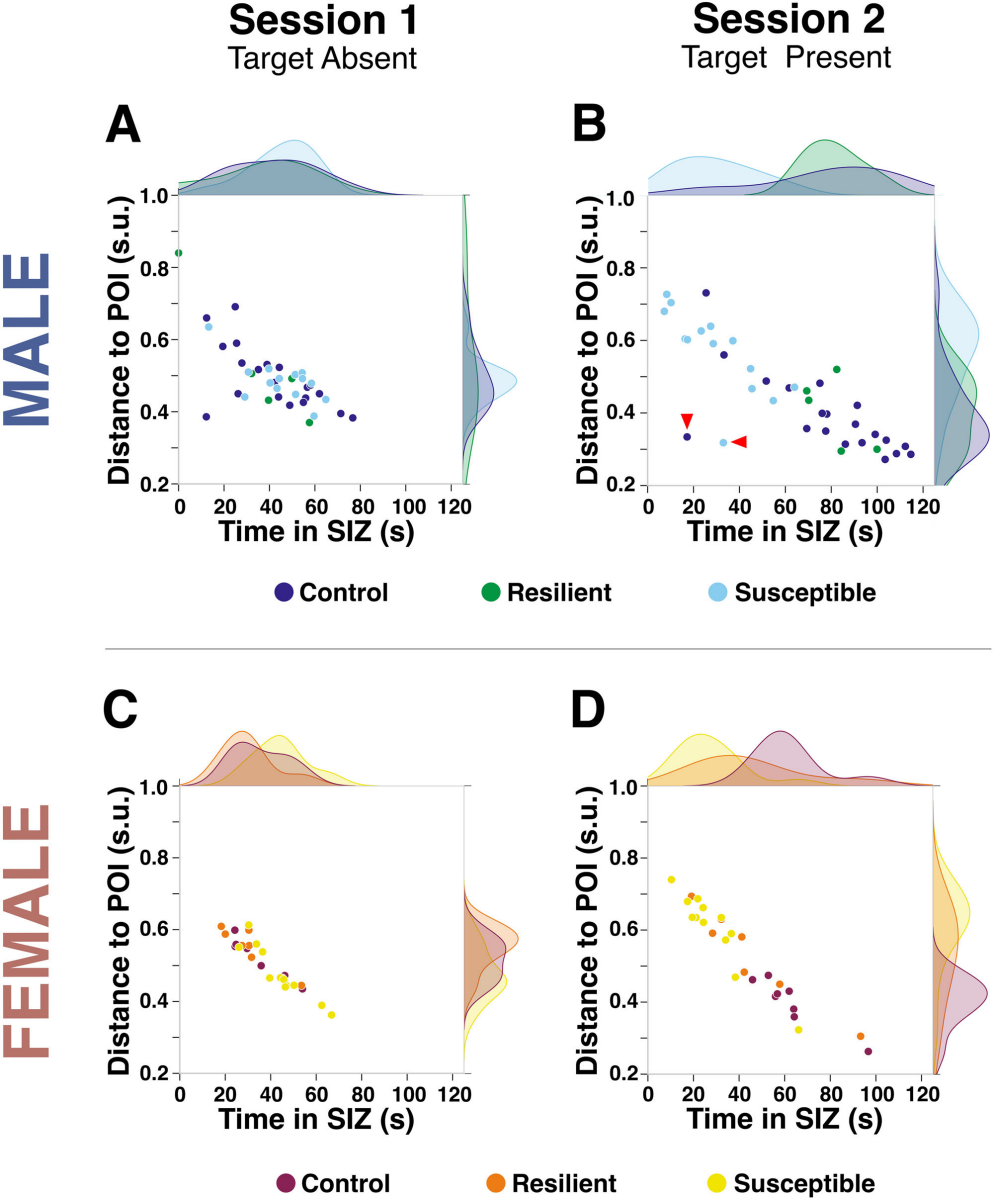
Individual differences in social interaction are revealed when combining time and distance metrics. Scatter plots showing average distance to the point of interest (POI) versus time spent in the social interaction zone (SIZ) for control, resilient, and susceptible mice. *Males*: **A** In Session 1, when the social target was absent, the data distribution across the 3 groups overlaps. **B** In Session 2, when the social target was present, there is less overlap in data distribution and large variability in social interaction behavior emerges in both control group and CSDS-exposed mice (see examples indicated with red arrow heads). *Females*: **C** In Session 1, when the social target was absent, the data distribution across the 3 groups overlaps. **D** In Session 2, when the social target was present, there is less overlap in data distribution (Supplementary Table 2).

To better capture within-group behavioral variability in Session 1 and in Session 2 of the SIT, we visualized the relationship between the time spent in the SIZ and the average distance to the POI. We plotted these data separately for Session 1 and Session 2 and for males and females (Fig. 4). Social behavior across mice was significantly different in Session 2 (when the CD1 mouse was present) compared to Session 1 (when the CD1 mouse was absent) and this was the case for males and females (Supplementary Table 2).

In males, we identified a control mouse and one that was classified as susceptible, based on the time-based interaction ratio, that in spite of remaining close to the POI in Session 2, spent very short time in the SIZ (Fig. 4B; see red arrows). This behavioral pattern, non-detectable when using time-based metrics alone, introduces ambiguity when interpreting the impact of CSDS on social interaction: it is not clear if the mouse is avoiding the social target or merely remaining nearby. Concordance and discrepancy between the two methods to segregate susceptible-like and resilient-like social phenotypes are graphically shown in Supplementary Figure 3. When using these two metrics to characterize behavioral phenotypes in control mice, we found one male showing a susceptible-like trait and another one showing an ambiguous social phenotype. This type of analysis also reveals large variability in social interaction behavior in the control group. By jointly examining distance and time, we can uncover such ambiguous cases, particularly when the female data suggest that classifying social interaction behavior using time-based interaction ratios may be more stable, although larger sample sizes are needed to explore this issue.

### Social Engagement Index integrates time and distance metrics and predicts self-care-like behavior

Given the complementary value of time and distance-based measures, we propose a new metric—the Social Engagement Index—which integrates both dimensions. The Social Engagement Index is calculated as the ratio of the time-based social interaction ratio to the distance-based ratio (see Eq. 5 in Materials and Methods section), providing a continuous measure that helps differentiate mice that seem to show ambiguous approach or avoidance phenotypes.

**Fig. 5:**
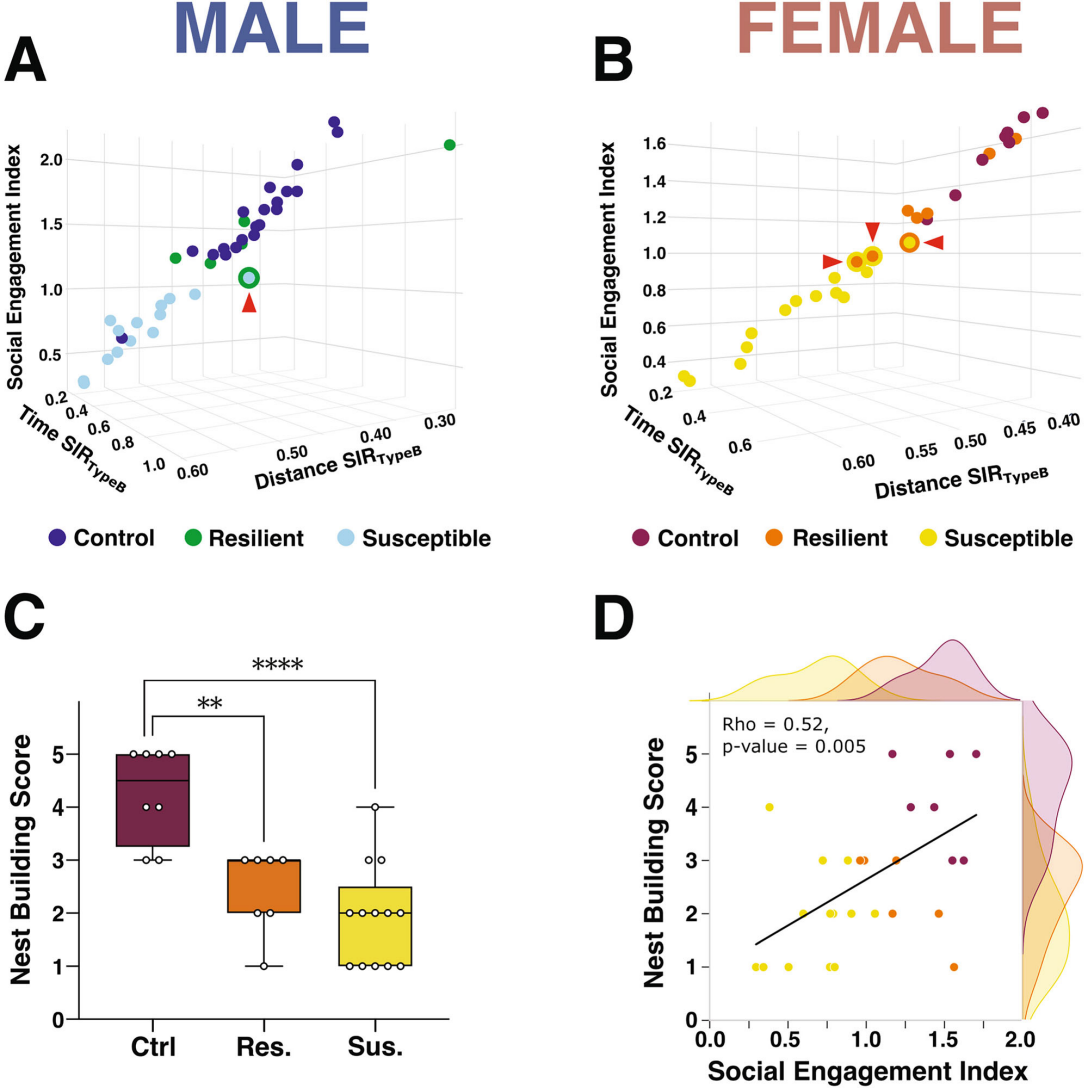
Social Engagement Index: integration of time and distance and prediction of self-care-like behavior. **A**, **B** 3D plots of the Social Engagement Index (SEI) as a function of time-based (Time SIRTypeB) and distance-based (Distance SIRTypeB) social interaction ratios in control, resilient, and susceptible mice. **A** In males, one susceptible mouse (green circle, red arrow) clustered with the resilient group (SEI > 1). **B** In females, two resilient mice (yellow circles) clustered with the susceptible group (SEI < 1), and one susceptible mouse (orange circle) aligned with the resilient group—demonstrating how Social Engagement Index helps detect within-group variability in social profiles. **C** Nestlet building scores were significantly higher in control mice compared to both resilient and susceptible groups (Supplementary Table 3A), indicating reduced self-care behavior in socially stressed females. **D** Social Engagement Index was positively correlated with nestlet building scores (Supplementary Table 3B), suggesting that Social Engagement Index captures aspects of social defeat-related phenotypes. Bar graph is presented as mean values  ±  SEM. **p < 0.01, **** *p* < 0.0001.

To facilitate visualization, we developed a Python script using the Plotly package (*Output_plots_in_3D.py*, available in the https://github.com/madmaxpython/DeepOF_SIT/tree/main/3D_plots), which plots Social Engagement Index in relation to both time- and distance-based ratios. This interactive 3D visualization allows experimenters to explore individual behavioral profiles while simultaneously assessing global distribution patterns across experimental groups. As shown in Fig. 5A, B (see also Supplementary Materials), this approach reveals mice that fall outside their expected group cluster—e.g., susceptible mice aligning with resilient ones, and vice versa (see red arrow).

To further validate the relevance of the Social Engagement Index, we explored whether it could also reflect behavioral dimensions outside of direct social interaction, such as stress-induced impairments in self-care. To this end, we used data we obtained in the experiment with females for which we assessed nest building, a well-established measure of self-care, often impaired by chronic stress [22]. Nest quality differed significantly across control, resilient and susceptible groups (Fig. 5C), with control mice building higher quality nests than both resilient and susceptible groups. While the individual time and distance metrics correlate with nest building (see Supplementary Fig. 4), the Social Engagement Index, which also correlated significantly with the nest building score (Fig. 5D), provides better discrimination between social interaction and social avoidance traits. This indicates that this index not only captures subtleties in social interaction but can also be employed to predict the impact of CSDS on other behavioral domains. These findings highlight the potential of the Social Engagement Index as a multidimensional indicator of stress vulnerability, linking, for example, deficits in social avoidance and self-care.

## Discussion

This study introduces a refined analytical framework to characterize social behavior in mice following CSDS. We propose a continuous Social Engagement Index that incorporates not only the time spent in the interaction zone but also the mouse’s proximity to the social target. This multi-dimensional approach reveals nuanced behavioral phenotypes that may often missed by the traditional, binary classification of “susceptible” versus “resilient.” Our analysis identified mice that, while categorized as susceptible by conventional time-based metrics, demonstrated social engagement levels comparable to resilient animals when distance was factored in. Conversely, some mice traditionally classified as resilient exhibited social avoidance patterns similar to susceptible mice. This finding supports growing evidence that while traditional social interaction analyses are efficient, their simplicity may mask the complex nature of social behavior [7]. A key strength of our approach is its translational potential and accessibility. By leveraging open-source tools, we demonstrate that our proposed index can be easily replicated and adapted across different experiments and between male and female rodents. The principles of this framework could be readily used to analyze data derived from different social behavioral tests, such as the three-chamber social novelty test [23] or other social interaction tests [24], providing a more robust measure of social preference across different contexts. We found that the Social Engagement Index correlates with nest building, a measure of self-care behavior. Nest building is widely recognized as a robust measure of self-care in rodents, reflecting their motivation and ability to maintain a suitable living environment, and has been shown to be altered following chronic social defeat stress [25, 26] Impairments in nest building could resemble self-neglect observed in humans suffering from depressive disorders, particularly among older adults [27]. Thus, nest building serves not only as a behavioral readout of self-care but also as an indicator of broader motivational and affective deficits associated with chronic stress exposure. It will be interesting to assess whether the Social Engagement Index correlates with measures of anhedonia, such as the sucrose preference test, and with anxiety-like behaviors, as evaluated by the elevated plus maze and forced swim test [28, 29]. Integrating these behavioral assays will provide a more comprehensive understanding of the Social Engagement Index’s predictive value and its capacity to serve as a multidimensional indicator of stress vulnerability and resilience.

Our work aligns with a broader call within the neuroscience community for more ethologically rich assessments of social behavior [30]. Recent advances in machine learning-based software, such as DeepOF, Keypoint-Moseq [31], and SimBA [32], are making these detailed analyses more feasible. The simplicity of the Social Engagement Index allows this metric to be used across the scientific community with minimal computational overhead, promoting standardization across labs with varying technical resources. While the current index adds the crucial dimension of distance, the clear next step is to leverage these tools to deconstruct social interaction into its constituent elements. This will allow us to differentiate between active investigation (e.g., head/nose-point tracking toward the CD-1) versus passive proximity, and to quantify subtle defensive postures (e.g., stretched or flattened body positions) that provide an even deeper characterization of the animal’s affective state. The widespread adoption of such multi-dimensional approaches, fostered by open-source collaboration, is essential for developing new standards that ensure that preclinical procedures are reliable and reproducible [33, 34].

Refining behavioral analysis derived from preclinical models is important to eventually be able to extrapolate findings from rodent studies to human psychiatric conditions. The misclassification of rodent behavioral phenotypes can have detrimental consequences, for example it may cause potential promising therapeutic targets to be overlooked. By providing a more accurate, individualized behavioral profile that transcends a simple binary outcome, our study addresses this challenge, highlighting that individual variability is a core component of the stress response. Recognizing the intricate landscape of social behaviors impacted an adverse event can help developing tailored interventions for stress-related disorders, as individual differences are key predictors of susceptibility and treatment response [35, 36]. By adopting and advancing such quantitative methodologies, we contribute to a collective effort to deepen our understanding of psychiatric disorders [15, 34, 37–39].

## Supplementary information

Supplementary figures and tables

## Data Availability

The experimental data that support the findings of this study are available in http://github.com/madmaxpython/DeepOF_SIT.
