# Supplementary material for: Classification of social behavioral responses in stress and non-stress adult male mice with high precision
Source: NPP Digit Psychiatry Neurosci. 2025 Dec 3;3:29. doi: 10.1038/s44277-025-00047-8 (PMC12673097; doi:10.1038/s44277-025-00047-8)
Supplement: Supplementary file 1 — Supplementary figures and tables [file 44277_2025_47_MOESM1_ESM.pdf]

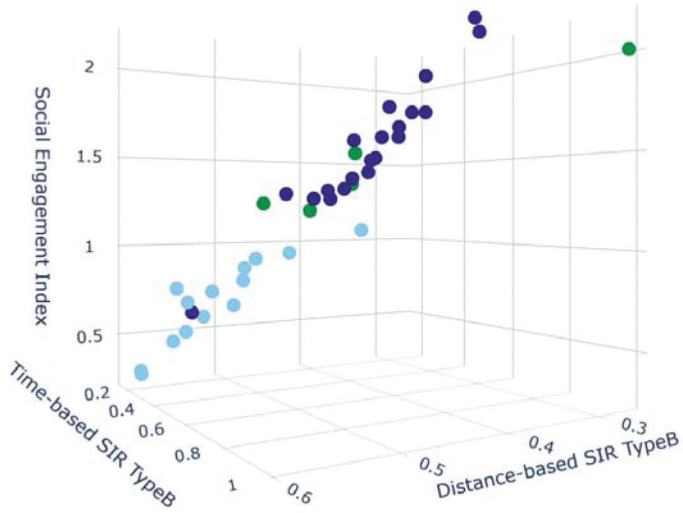

Visit (https://github.com/madmaxpython/DeepOF_SIT/tree/main/3D_plots) to download interactive plot

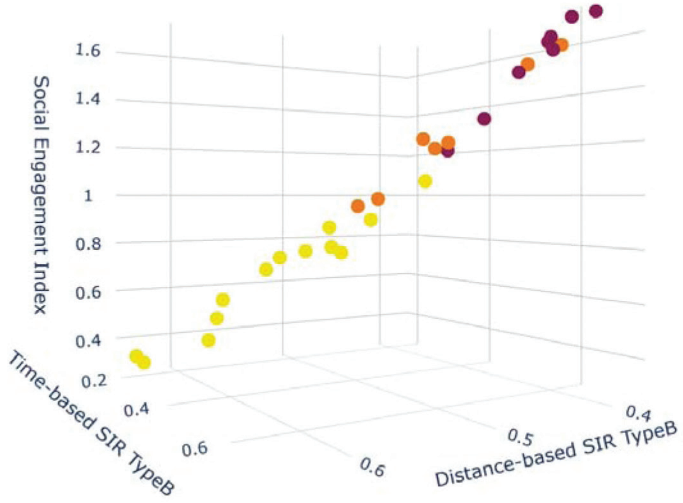

Visit (https://github.com/madmaxpython/DeepOF_SIT/tree/main/3D_plots) to download interactive plot

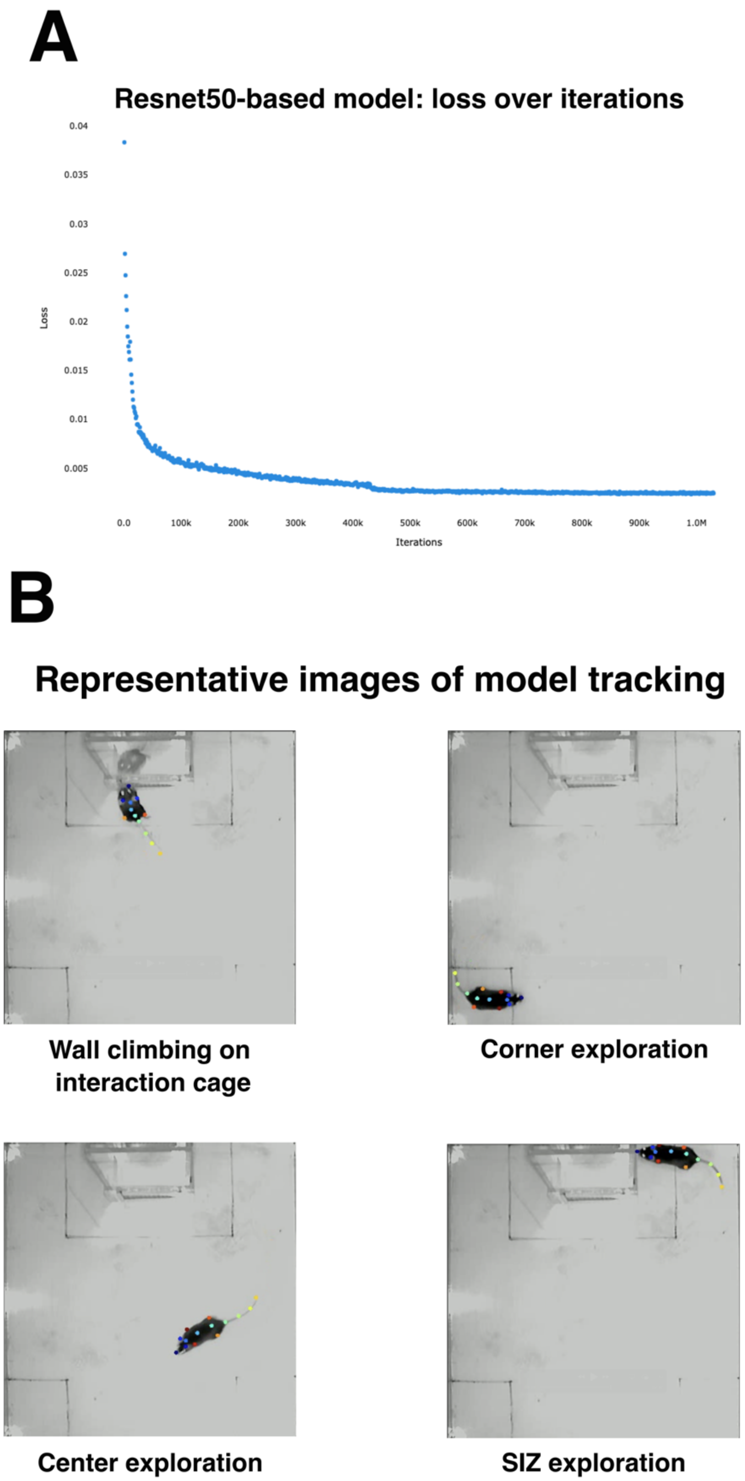

Supplementary Figure 2. Training and performance of the DeepLabCut model used for tracking social interaction in mice.

A. Training loss curve showing progressive learning, with loss plateauing around 500,000 iterations, indicating convergence of the model.

B. Example frames illustrating accurate tracking body parts of mice across various behavioral contexts: wall climbing, corner exploration, center exploration, and social interaction zone (SIZ).

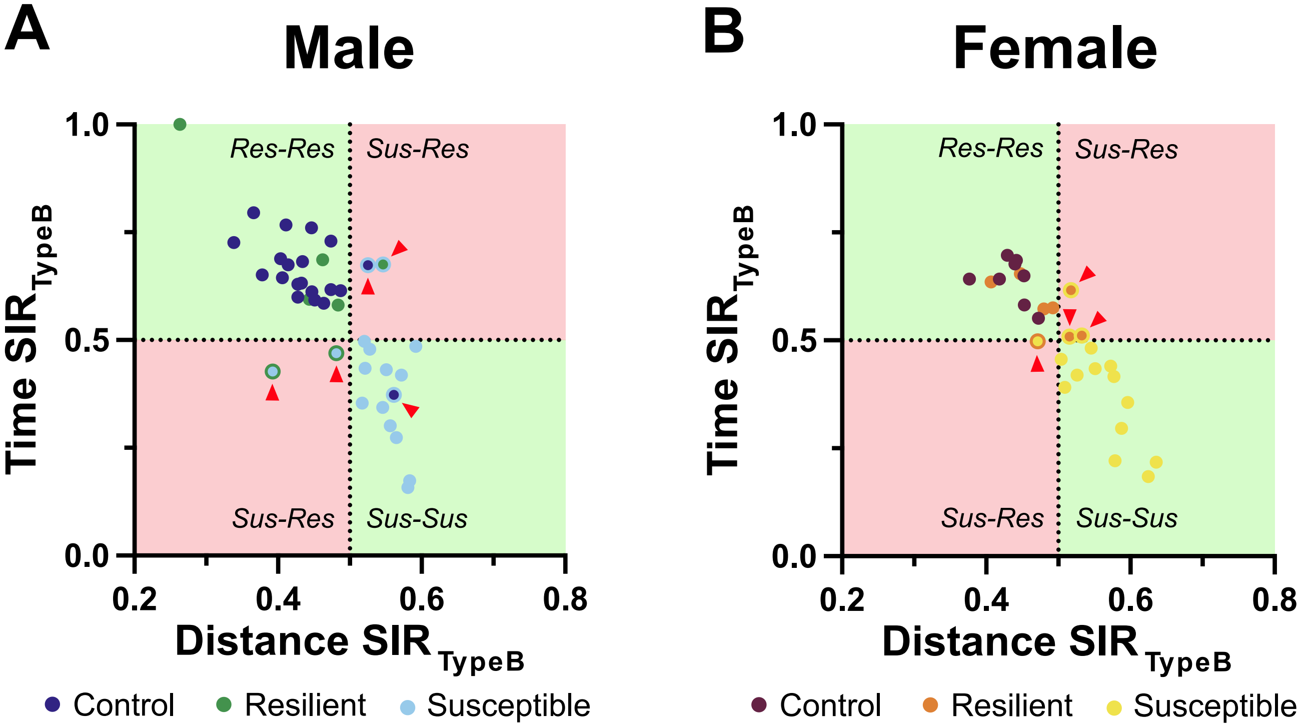

Supplementary Figure 3. Concordance between time-based and distance-based social interaction ratio (SIR) classifications. Four-quadrants scatter plots comparing time-based SIR (y-axis) and distance-based SIR (x-axis) measurements in male (A) and female (B) mice. Quadrants defined by threshold lines at 0.5 represent classification concordance: Res-Res (lower-left) and Sus-Sus (upper-right) indicate agreement between methods, while Sus-Res quadrants (upper-left and lower-right) represent discordant classifications between time-distance. Red arrowheads highlight individual mice where time-based and distance-based SIR measurements yield conflicting resilient/susceptible classifications, with circled color representing shift in classification based on the distance SIR. When using these two metrics to characterize behavioral phenotypes in control mice, we found one male showing a susceptible -like trait and another one showing an ambiguous social phenotype.

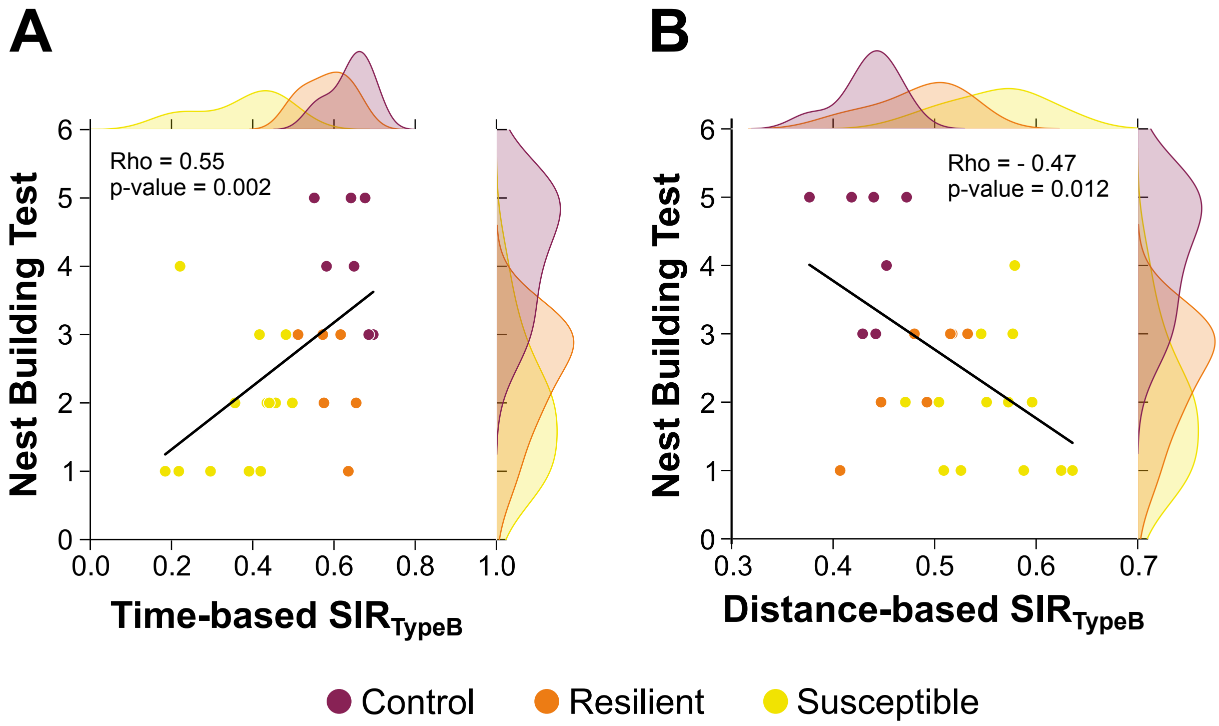

Supplementary Figure 4. Nest Building Score correlates with both time-based and distance-based social interaction ratios. Correlation analysis between Nest Building Test scores and social interaction ratios for (A) time-based SIR_{TypeB} (Rho = 0.55, p-value = 0.002) and (B) distance-based SIR_{TypeB} (Rho = -0.47, p-value = 0.012). Each data point represents an individual mouse, with colors corresponding to experimental groups. Marginal density plots show the distribution of values for each measure.

Table 1 Detailed statistics for Fig. 3

	Statistical test	Factor	n	Statistic	95% confidence interval	*p* value (adjusted where appropriate)	Corresponding figure	Sex
**A**	Two-way ANOVA	Interaction	Control = 20<br>Stress = 19	F (1, 74) = 11.32		**0.0012**	**Fig. 3A**	**MALE**
		Session		F (1, 74) = 11.97		**0.0009**		
		Stress		F (1, 74) = 10.12		**0.0021**		
	Šídák's multiple comparisons test	Session 1 vs Session 2 - within Control		t(74) = 4.889	-53.61 to -19.48	**<0.0001**		
	Šídák's multiple comparisons test	Session 1 vs Session 2 - within Stress		t(74) = 0.065	-18.01 to 17.00	0.9973		
**B**	Two-way ANOVA	Interaction	Control = 20<br>Stress = 19	F (1, 74) = 7.066		**0.0096**	**Fig. 3B**	
		Session		F (1, 74) = 2.131		0.1486		
		Stress		F (1, 74) = 8.099		**0.0057**		
	Šídák's multiple comparisons test	Session 1 vs Session 2 - within Control		t(74) = 2.950	0.02294 to 0.1800	**0.0085**		
	Šídák's multiple comparisons test	Session 1 vs Session 2 - within Stress		t(74) = 0.837	-0.1101 to 0.05102	0.6465		
**C**	Ordinay one-way ANOVA	Group	Control = 20<br>Resilient = 5<br>Susceptible = 14	F (2, 36) = 31.62		**<0.0001**	**Fig. 3C**	
	Tukey's multiple comparisons test	Control vs. Res.		t(36) = 1.422	-0.1891 to 0.07887	0.5783		
	Tukey's multiple comparisons test	Control vs. Sus.		t(36) = 10.28	0.1843 to 0.3710	**<0.0001**		
	Tukey's multiple comparisons test	Res. vs. Sus.		t(36) = 8.239	0.1932 to 0.4724	**<0.0001**		
**D**	Ordinay one-way ANOVA	Group	Control = 20<br>Resilient = 5<br>Susceptible = 14	F (2, 36) = 11.73		**0.0001**	**Fig. 3D**	
	Tukey's multiple comparisons test	Control vs. Res.		t(36) = 0.060	-0.07495 to 0.07238	0.999		
	Tukey's multiple comparisons test	Control vs. Sus.		t(36) = 6.580	-0.1491 to -0.04638	**0.0001**		
	Tukey's multiple comparisons test	Res. vs. Sus.		t(36) = 4.343	-0.1732 to -0.01968	**0.011**		

**E**	Two-way ANOVA	Interaction	Control = 8 Stress = 15	F (1, 21) = 16.85		**0.0005**	**Fig. 3E**	**FEMALE**
		Session		F (1, 21) = 10.30		**0.0042**		
		Stress		F (1, 21) = 3.364		0.0808		
	Šídák's multiple comparis ons test	Session 1 vs Session 2 - within Control		t(21) = 4.528	-40.86 to -12.49	**0.0004**		
	Šídák's multiple comparis ons test	Session 1 vs Session 2 - within Stress		t(21) = 0.704	-7.094 to 13.62	0.7045		
**F**	Two-way ANOVA	Interaction	Control = 8 Stress = 15	F (1, 21) = 19.78		**P=0.0002**	**Fig. 3F**	
		Session		F (1, 21) = 2.171		P=0.1554		
		Stress		F (1, 21) = 4.945		**P=0.0373**		
	Šídák's multiple comparis ons test	Session 1 vs Session 2 - within Control		t(21) = 3.666	0.03935 to 0.1900	**0.0029**		
	Šídák's multiple comparis ons test	Session 1 vs Session 2 - within Stress		t(21) = 2.521	-0.1126 to - 0.002593	**0.0393**		
**G**	Ordinay one-way ANOVA	Group	Control = 8 Resilient = 6 Susceptible = 9	F (2, 25) = 25.54		**<0.0001**	**Fig. 3G**	
	Tukey's multiple comparis ons test	Control vs. Re s.		t(25) = 2.640	- 0.02508 to 0.1663	0.1743		
	Tukey's multiple comparis ons test	Control vs. Su s.		t(25) = 9.835	0.1506 to 0.3228	**<0.0001**		
	Tukey's multiple comparis ons test	Res. vs. Sus.		t(25) = 6.362	0.07268 to 0.2595	**0.0006**		
**H**	Ordinay one-way ANOVA	Group	Control = 8 Resilient = 6 Susceptible = 9	F (2, 25) = 22.24		**<0.0001**	**Fig. 3H**	
	Tukey's multiple comparis ons test	Control vs. Re s.		t(25) = 3.145	- 0.1041 to 0.00589 7	0.0866		
	Tukey's multiple comparis ons test	Control vs. Su s.		t(25) = 9.189	-0.1723 to - 0.07680	**<0.0001**		
	Tukey's multiple comparis ons test	Res. vs. Sus.		t(25) = 5.336	-0.1253 to - 0.02564	**0.0025**		

Table 2 Detailed statistics for Fig. 4

	Statistical test	Factor	n	Statistic	Wilks' Λ	*p* value	Corresponding figure	Sex
**A**	MANOVA	Session 1: Control vs Resilient vs Susceptible	Control = 20 Resilient =5 Susceptible = 14	F(4,70) = 0.314	0.965	0.8678	**Fig. 4A**	**MALE**
**B**	MANOVA	Session 2 : Control vs Resilient vs Susceptible		F(4,70) = 7.917	0.4741	**<0.0001**	**Fig. 4B**	
**C**	MANOVA	Session 1: Control vs Resilient vs Susceptible	Control = 8 Resilient = 6 Susceptible = 9	F(4,48) = 1.799	0.756	0.1445	**Fig. 4C**	**FEMALE**
**D**	MANOVA	Session 2:Control vs Resilient vs Susceptible		F(4,48) = 4.844	0.508	**0.0023**	**Fig. 4D**	

	Table 3 Detailed statistics for Fig. 5							
	**Statistical test**	**Factor**	**n**	**Statistic**	**95% confidence interval**	***p* value (adjusted where appropriate)**	**Corresponding figure**	**Sex**
**A**	Ordinary one-way ANOVA	Group	Control = 8 Resilient = 6 Susceptible = 9	F(2, 25) = 17.12		**<0.0001**	**Fig. 5C**	**FEMALE**
	Tukey's multiple comparisons test	Control vs. Res.		t(25) = 5.546	0.6644 to 2.978	**0.0017**		
	Tukey's multiple comparisons test	Control vs. Sus.		t(25) = 8.160	1.322 to 3.331	**<0.0001**		
	Tukey's multiple comparisons test	Res. vs. Sus.		t(25) = 1.699	-0.5425 to 1.554	0.4634		
**B**	Spearman's rank correlation	SEI and Nest Building score	Control = 8 Resilient = 6 Susceptible = 9	rhô = 0.517	0.1219 to 0.8098	**0.0049**	**Fig. 5D**	
